# Improvement of quality of life in elderly after inguinal hernioplasty

**DOI:** 10.1186/1471-2318-11-S1-A44

**Published:** 2011-08-24

**Authors:** Rodalia Patti, Paolo Aiello, Gaetano Di Vita

**Affiliations:** 1 Department of Surgical and Oncological Science, University of Palermo, Italy

## Background

Inguinal hernia represents one of the most common diseases in the elderly. It causes aching and unsightly swelling affecting quality of life (QOL), with a concomitant risk of strangulation. Despite this, an assumption of the risks of surgery may lead clinicians, particularly non-surgeons, to advise elderly patients against the repair of hernia, especially if asymptomatic [1]. The aim of this study was to evaluate QOL by a short Form 36 (SF-36) questionnaire [2] in elderly patients undergoing inguinal hernioplasty.

## Materials and methods

Forty male patients of an age ranging from 65 to 92 years affected by unilateral symptomatic inguinal hernia were included and divided into two groups . 15 patients were allocated to the first group who refused hernioplasty, whereas, 25 patients who underwent to elective Liechtenstein hernioplasty were included in the second group, using a high density polypropylene mesh, under local anaesthesia. The SF-36 questionnaire was administered to all patients (both surgically treated and untreated ones) included in the study before and 6 months after surgery. Global analyses of the 8 domains of SF-36 and 2 comprehensive indexes of SF-36, Physical Component Summary (PCS) and Mental Component Summary (MCS) were performed.

## Results

The two groups of patients were similar in age, type of hernia according to Nyhus, duration of disease and anaesthesiologic risk according to ASA and Barthel and Charlson index. Liechtenstein hernioplasty caused no major complications. One patient from the untreated group underwent emergency surgery for hernia strangulation. All 8 domains of SF-36 and MCS and PCS scores improved remarkably within 6 months after the surgery in patients who underwent inguinal hernioplasty. In the group of patients who received no surgical treatment no significant differences were detected.

## Conclusions

Inguinal hernioplasty under local anaesthesia is a safe procedure for elderly patients affected by symptomatic inguinal hernia. It improves the QOL and therefore represents a clear cut indication for elective hernia repair in elderly patients.
